# Association Between Cachexia Defined by the Asian Working Group for Cachexia Criteria and One-Year Post-Discharge Mortality in Older Adults with Acute Decompensated Heart Failure

**DOI:** 10.3390/geriatrics11040098

**Published:** 2026-08-04

**Authors:** Daisuke Suzuki, Toshimi Sato, Yuichiro Sasamoto, Shun Monoe, Keita Kaneko, Shinichiro Morishita

**Affiliations:** 1 Department of Rehabilitation, Southern Tohoku General Hospital, Koriyama 963-8563, Japan; mh258517@fmu.ac.jp; 2 Department of Physical Therapy, Graduate School of Health Sciences, Fukushima Medical University, Fukushima 960-8516, Japan; mh258523@fmu.ac.jp (S.M.); mh258510@fmu.ac.jp (K.K.); 3 Department of Physical Therapy, School of Health Sciences, Fukushima Medical University, Fukushima 960-8516, Japan; ssmty59675522@outlook.jp; 4 Department of Rehabilitation, Ohta Nishinouchi Hospital, Ohta General Hospital Foundation, Koriyama 963-8558, Japan; 5 Department of Rehabilitation, Wakamatsu Aoi Clinic, Aizuwakamatsu 965-0840, Japan; 6 Department of Rehabilitation, Hoshi General Hospital, Koriyama 963-8501, Japan

**Keywords:** cachexia, heart failure, older adults, mortality, Asian working group for cachexia

## Abstract

**Background**: Cachexia is a well-known clinically important condition in older adults with heart failure, but evidence regarding cachexia defined by the Asian Working Group for Cachexia (AWGC) criteria in those with acute decompensated heart failure (ADHF) remains limited. **Methods**: This prospective observational study included 78 patients aged 65 years or older who were hospitalized for ADHF. Cachexia on admission was diagnosed using the AWGC criteria. The primary outcome was all-cause mortality within one year after discharge and the secondary outcome was all-cause readmission within the same period. **Results**: Cachexia was diagnosed in 26 patients (33.3%). Compared with the patients without cachexia, those with cachexia were older and had a lower body mass index, poorer nutritional status, and reduced physical function. Cachexia was associated with a significantly higher risk of all-cause mortality after discharge. In the adjusted Cox proportional hazards model, cachexia remained significantly associated with all-cause mortality within one year after discharge (HR, 3.04), whereas no significant association was observed with all-cause readmission. **Conclusions**: Among older adults hospitalized for ADHF, AWGC-defined cachexia on admission was associated with increased all-cause mortality within one year after discharge. Although these findings suggest that AWGC-defined cachexia may be useful for identifying a clinically vulnerable phenotype, they should be interpreted as exploratory and confirmed in larger multicenter studies.

## 1. Introduction

The number of patients with heart failure in Japan is expected to continue to increase as the population ages, and to reach approximately 1.3 million by 2030. Consequently, the burden of heart failure is likely to remain substantial, particularly among older adults, raising concerns about a so-called “heart failure pandemic.” [1]. Patients with heart failure often have multiple comorbidities, which not only impair physical function and reduce quality of life, but also increase mortality [2].

Cachexia is a metabolic syndrome associated with chronic disease and characterized by nutritional deficiency, inflammation, oxidative stress, hypogonadism, anemia, insulin resistance, and increased protein catabolism, resulting in the loss of both skeletal muscle and adipose tissue. Clinically, it manifests as weight loss, muscle weakness, and reduced physical performance, and is recognized as an important predictor of poor outcomes in patients with chronic diseases [3]. This syndrome is also a major concern in heart failure, where its development is further accelerated by chronic neurohumoral activation and the aforementioned metabolic disturbances [4]. Previous studies reported the prevalence of cachexia in heart failure to range from approximately 8% to 42% [2], with cachexia being associated with increased mortality and impaired physical function [5].

Older adults with heart failure may be particularly susceptible to cachexia because of age-related sarcopenia, frailty, and malnutrition [6,7]. In this population, progression of cachexia may be associated with impaired physical function, difficulties in activities of daily living, and poor prognosis [7,8]; however, few studies have investigated the prevalence and clinical significance of cachexia in older adults hospitalized for acute decompensated heart failure (ADHF).

Several diagnostic criteria for cachexia have been proposed, among which the definition by Evans et al. has been widely used [3]. According to this definition, cachexia is diagnosed as weight loss or low body mass index (BMI) in addition to at least three of the following: muscle weakness, loss of appetite, fatigue, reduced lean body mass, and abnormal biochemistry such as inflammation or anemia. However, these criteria were developed primarily for Western populations and may not reflect the body composition and nutritional characteristics of Asian populations. In 2023, the Asian Working Group for Cachexia (AWGC) proposed diagnostic criteria specifically targeted at Asian populations. Ref. [9] defined cachexia on the basis of weight loss or low BMI in combination with loss of appetite, muscle weakness, and high inflammatory marker levels. The AWGC criteria may be particularly relevant for older Asian patients with heart failure because they incorporate thresholds and diagnostic components that reflect the body composition and nutritional characteristics of Asian populations. Compared with older definitions derived mainly from Western populations, the AWGC framework may provide a clinically applicable approach for identifying cachexia-related vulnerability in Asian geriatric cohorts. However, the acute hospitalization setting may modify the meaning of individual diagnostic components because body weight and BMI can be influenced by congestion and fluid overload, appetite-related symptoms may be affected by gastrointestinal congestion, and CRP may reflect both chronic inflammation and transient acute-phase responses. Therefore, the present study pragmatically applied the AWGC criteria to identify a heart-failure-associated cachexia phenotype on admission in older adults hospitalized for ADHF and examined its association with one-year post-discharge outcomes.

## 2. Materials and Methods

### 2.1. Study Flow

In this prospective observational study, 89 patients aged 65 years or older who were hospitalized for ADHF at Southern Tohoku General Hospital (Koriyama, Japan) between August 2021 and March 2022 were screened, and 78 were included in the analysis. The inclusion criteria were hospitalization for ADHF, prescription of physical therapy by a physician, age ≥65 years, and independence in out-of-bed activities prior to hospitalization. The exclusion criteria were missing data required for the diagnosis of cachexia and in-hospital death.

The protocol for this study was approved by the Ethics Committee of Southern Tohoku General Hospital (Approval No. 508). Consent to participate in the present study was obtained through an opt-out approach. Study information was posted on the hospital website and patients were given the opportunity to decline participation.

### 2.2. Data Collection and Definitions

#### 2.2.1. Baseline Characteristics

Baseline variables included age, sex, BMI, comorbidities, long-term care insurance certification status, medication use, New York Heart Association (NYHA) functional class, history of hospitalization for heart failure, echocardiographic findings, including left ventricular ejection fraction, and laboratory data including hemoglobin, albumin, C-reactive protein (CRP), creatinine, blood urea nitrogen (BUN), and estimated glomerular filtration rate (eGFR). NYHA functional class was categorized as class III/IV. All baseline data were obtained from electronic medical records.

#### 2.2.2. Diagnosis of Cachexia

In this study, cachexia was operationally diagnosed according to the AWGC criteria and interpreted as a heart-failure-associated cachexia phenotype in older adults hospitalized for ADHF, rather than as an etiology-specific diagnosis of ‘cardiac cachexia.’ The underlying chronic disease criterion of the AWGC framework was considered to be fulfilled by the presence of heart failure requiring hospitalization for ADHF.

The weight-related criterion was considered fulfilled when patients had either documented body weight loss >2% during the preceding 3–6 months or BMI < 21 kg/m^2^ on admission. Information on recent weight loss was obtained from medical records and nutritional assessment documentation. Because body weight and BMI on admission may be affected by congestion and fluid overload in ADHF, these variables were interpreted as pragmatic clinical indicators rather than as strict measures of tissue wasting.

Loss of appetite was not assessed using a validated appetite-specific questionnaire. Instead, it was operationally identified from the SGA [10] interview documentation on admission. Specifically, patients were classified as having appetite loss when the medical history section of the SGA indicated reduced oral intake or gastrointestinal symptoms suggestive of reduced appetite. This approach was based on structured nutritional assessment documentation but has not been validated as a substitute for an appetite-specific instrument.

Handgrip strength was measured within two days of admission as part of the physical function assessment. Low handgrip strength was defined according to the AWGC cut-off values of <28 kg for men and <18 kg for women. Handgrip strength was measured using a digital handgrip dynamometer (TKK5401, Takei Scientific Instruments Co., Ltd., Niigata, Japan). Measurements were performed twice for each hand in the seated position, and the maximum value was used for analysis.

CRP was measured from blood samples obtained on admission, and CRP > 0.5 mg/dL was considered elevated according to the AWGC criteria. AWGC-defined cachexia was diagnosed when patients had an underlying chronic wasting disease, fulfilled the weight-related criterion, and met at least one of the following criteria: appetite loss, low handgrip strength, or elevated CRP.

### 2.3. Outcomes

The primary outcome was all-cause mortality within one year after discharge, and the secondary outcome was all-cause readmission within the same period. In the present study, readmission was defined as the time to first all-cause readmission. Follow-up data for mortality and readmission were obtained through mail surveys and review of medical records.

### 2.4. Statistical Analysis

Continuous variables are presented as medians and interquartile ranges (IQRs), and categorical variables as counts and percentages. Between-group comparisons were performed using the Mann–Whitney *U* test for continuous variables and the chi-square test or Fisher’s exact test for categorical variables.

The proportional hazards assumption was assessed using log-minus-log survival plots and by testing the interaction between cachexia status and survival time.

For the primary analysis, a Cox proportional hazards model was used to examine the association between cachexia and all-cause mortality within one year of discharge, and hazard ratios (HRs) with 95% confidence intervals (CIs) were calculated. Age, sex, and BNP were included as adjustment variables. Covariates for the multivariable Cox model were selected a priori based on clinical relevance and the limited number of mortality events. Age and sex were included as fundamental demographic variables, and BNP was included as a marker of heart failure severity. BNP was log-transformed because of its skewed distribution. Follow-up was censored 365 days after discharge. Kaplan–Meier curves were also constructed, and between-group differences were assessed using the log-rank test. The association between cachexia and time to first all-cause readmission within one year after discharge was evaluated in the same manner as the secondary analysis.

All statistical analyses were performed using SPSS version 30 (SPSS Inc., Chicago, IL, USA) and a two-sided *p*-value < 0.05 was considered significant.

## 3. Results

### 3.1. Study Flow and Baseline Characteristics

**Figure 1:**
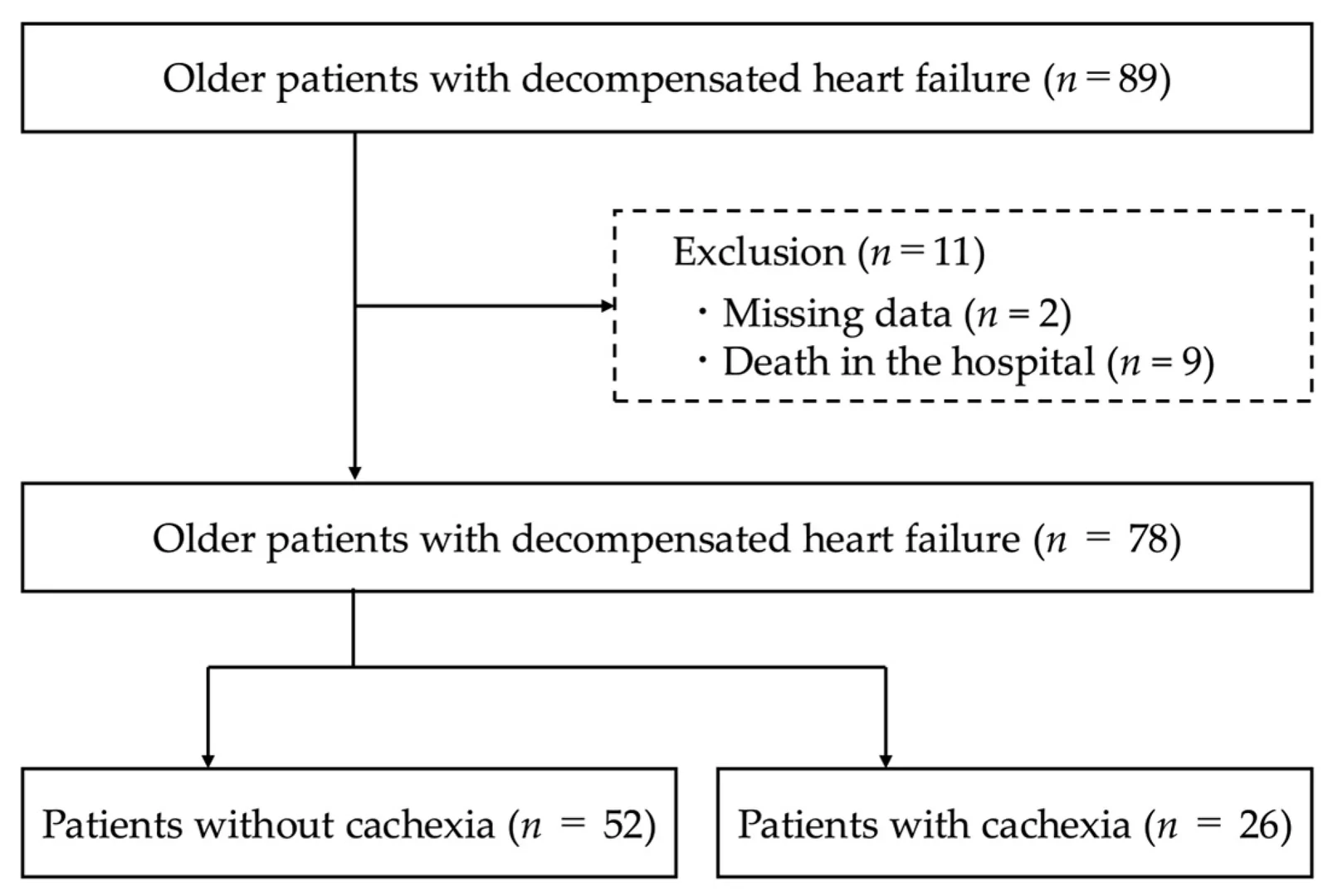
Study flow chart.

Of the 89 patients aged 65 years or older who were hospitalized for heart failure, 11 were excluded because cachexia could not be diagnosed, due to missing data required for the diagnosis (*n* = 2) or because of in-hospital death (*n* = 9). The final analysis included 78 patients (Figure 1). No imputation was performed for missing data. Patients were excluded from the primary analysis only when cachexia status could not be determined, because essential data for the AWGC diagnosis were unavailable. For variables not required for cachexia diagnosis, the number and percentage of missing values are shown in Table 1. Missingness for each AWGC component is shown in Supplementary Table S1. The median age was 85 years (IQR: 80–90.5), and 57.7% of the patients were male. The median BNP level was 547 pg/mL, and the rate of heart failure with reduced ejection fraction was 36.8%.

Cachexia was diagnosed in 26 patients (33.3%). Compared with patients without cachexia, those with cachexia were older and had higher rates of dyslipidemia and hospitalization for heart failure within the previous six months. The cachexia group also had a lower BMI, albumin and hemoglobin levels, and grip strength, as well as smaller arm and calf circumferences.

### 3.2. Association Between Cachexia and Clinical Outcomes

**Figure 2:**
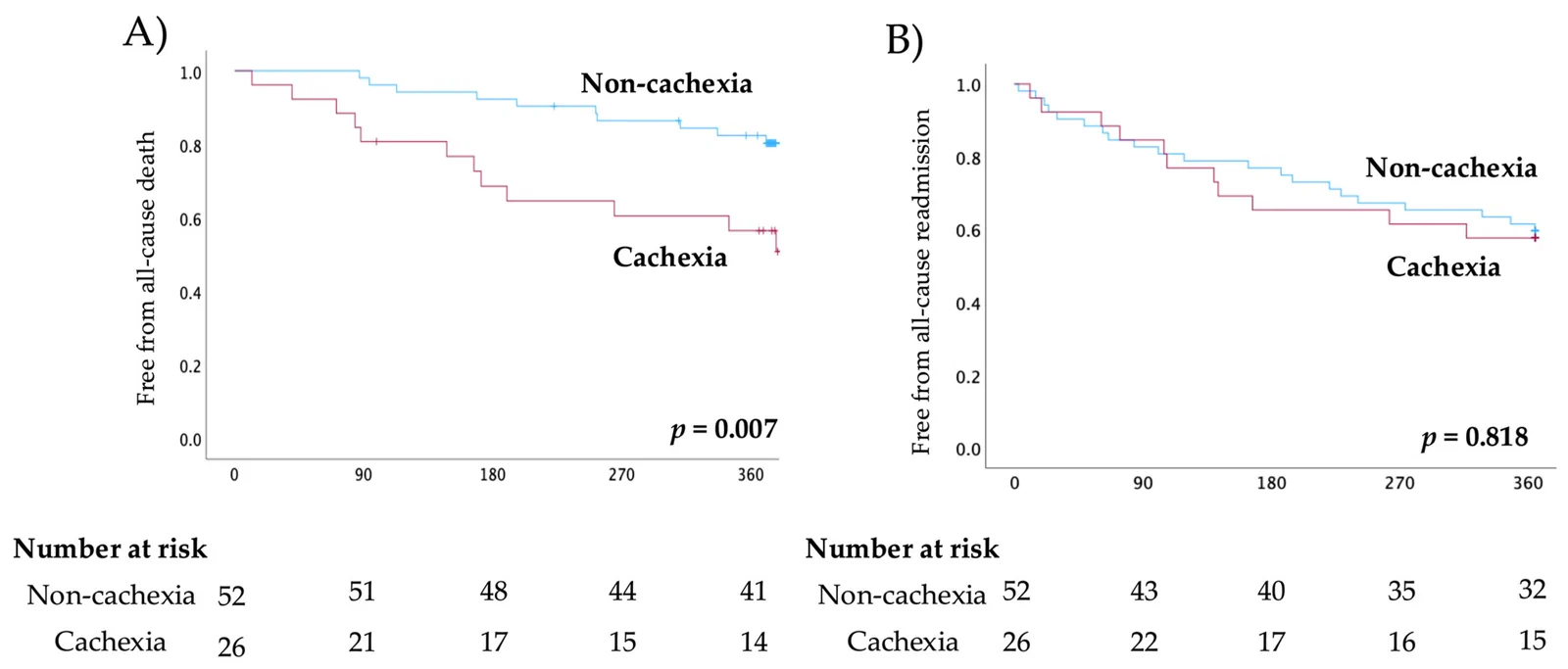
Kaplan–Meier curves for adverse clinical events within one year after discharge in the cachexia and non-cachexia groups. (**A**) All-cause mortality within one year after discharge; (**B**) time to first all-cause readmission within one year after discharge.

Assessment of the proportional hazards assumption did not indicate a meaningful violation for the mortality or readmission models. The mean follow-up period after discharge was 323 days. During the first year after discharge, 23 patients (29.5%) died: 12 patients (46.2%) in the cachexia group and 11 (21.2%) in the non-cachexia group. Kaplan–Meier analysis revealed a higher incidence of all-cause mortality in the cachexia group (log-rank *p* = 0.007) (Figure 2). In the adjusted Cox proportional hazards model, cachexia remained significantly associated with all-cause mortality within one year after discharge (HR, 3.04; 95% CI, 1.25–7.41; *p* = 0.014) (Table 2).

For the secondary endpoint, 32 patients (41.0%) experienced at least one all-cause readmission within one year after discharge: 11 patients (44.0%) in the cachexia group and 21 (40.1%) in the non-cachexia group. No significant difference was observed between the two groups on Kaplan–Meier analysis (log-rank *p* = 0.818) (Figure 2). Likewise, no significant association was observed between cachexia and all-cause readmission in the adjusted Cox proportional hazards model (HR, 1.65; 95% CI, 0.74–3.71; *p* = 0.223) (Table 3).

## 4. Discussion

This study investigated whether cachexia, defined by AWGC criteria at the time of admission, could identify a clinically vulnerable heart failure-related phenotype associated with one-year post-discharge mortality in elderly patients hospitalized for ADHF. As a result, the prevalence of cachexia was 33.3%, and cachexia diagnosed on admission was associated with higher all-cause mortality within one year of discharge.

A previous study reported that the prevalence rate of cachexia in heart failure ranges widely, from approximately 8% to 42% [2], but the study included only patients aged 60 to 75 years and did not include geriatric patients. In addition, because the study primarily focused on patients in a relatively stable clinical condition and the diagnosis of cachexia relied mainly on weight loss, it may not have fully captured cases characterized by muscle wasting or inflammation. Therefore, these prevalence estimates should be interpreted with caution. In contrast, Emi et al. [8], using the more comprehensive diagnostic criteria for cachexia proposed by Evans et al. [3], reported a prevalence rate of 35.5% at discharge among older patients hospitalized for heart failure. Furthermore, studies applying the AWGC diagnostic criteria have also been recently reported. Noda et al. [11] and Hashimoto et al. [12] examined the prevalence of cachexia and its association with prognosis in older patients with heart failure; however, both studies mainly included patients in the chronic phase or those who were relatively clinically stable. Accordingly, the reported prevalence rate of cachexia varies widely, ranging from approximately 20% to 70%, and this variation may reflect differences in study populations, timing of assessment, and diagnostic criteria. In this context, the present study is distinct in that it focused on patients hospitalized for ADHF and assessed cachexia on admission during the acute phase. Taken together, differences in the reported prevalence rates may be attributable to variations in disease stage, timing of assessment, and diagnostic criteria across study populations.

In ADHF, body weight and BMI on admission may be influenced by congestion and fluid retention, which can make nutritional assessment difficult [13]. Extracellular fluid volume is often markedly increased in ADHF and may change substantially in response to treatment [14]. In addition, changes in body weight during hospitalization do not necessarily correspond directly to changes in fluid balance [15]. As such, nutritional assessment based solely on body weight during the acute phase has important limitations. Although the definition proposed by Evans et al. [3] also emphasizes the importance of excluding fluid retention when evaluating weight loss, patients diagnosed with cachexia using the AWGC criteria on admission in the present study may still represent a subgroup with more advanced underlying wasting despite the influence of acute fluid overload. Thus, cachexia identified on admission may reflect a clinically susceptible phenotype characterized by systemic wasting and greater disease severity.

Patients in the cachexia group were also more likely to have a recent history of hospitalization for heart failure. Re-hospitalization for heart failure may reflect more advanced disease, chronic inflammation [16], persistent neurohumoral activation [17], and poorer nutritional status [18], all of which may be associated with ongoing catabolism and progressive loss of muscle and body mass. Accordingly, cachexia diagnosed on admission may reflect not only the current acute episode but also a longer-standing catabolic condition associated with disease progression. From a clinical perspective, assessment of cachexia on admission may aid in identifying patients with marked wasting during acute exacerbation of heart failure and assist in early risk stratification.

In the present study, cachexia diagnosed using the AWGC criteria was associated with all-cause mortality within one year of discharge. Cachexia in heart failure may reflect a systemic catabolic state characterized by chronic inflammation and neurohumoral activation [4] which may be linked to reduced skeletal muscle mass, impaired physical function, frailty, and sarcopenia [4,19]. Moreover, handgrip strength, one of the diagnostic components for cachexia, has been reported to be a predictor of early mortality in patients with ADHF, suggesting the clinical importance of evaluating muscle strength during the acute phase [20]. In addition, loss of muscle mass may be related to reduced respiratory muscle performance and poorer exercise tolerance. Furthermore, cachexia is associated with impaired immune function, which increases the risk of infection and reduces tolerance to acute physiological stress due to decreased systemic metabolic reserve [4,21,22]. As cachexia is a systemic disease accompanied by multi-organ dysfunction and increased catabolism at the organ level, including the myocardium, it may promote further deterioration of cardiac function [23]; therefore, cachexia is considered to increase the risk of death through multifaceted mechanisms [24]. Cachexia and weight loss in patients with heart failure are well-supported independent risk factors for mortality [5,25], consistent with the findings of this study.

In contrast, no clear association was observed between cachexia and all-cause readmission within one year of discharge. The lack of association between cachexia and readmission should be interpreted cautiously. In the present study, readmission was analyzed as the time to first all-cause readmission, which may underrepresent the recurrent and multifactorial nature of rehospitalization after ADHF. Readmission can be influenced by outpatient management, medication optimization, social support, comorbidities, and healthcare access, in addition to nutritional and functional vulnerability [26,27]. Future studies with larger samples should consider recurrent-event models and cause-specific readmission outcomes.

This study has several limitations. First, the assessment of appetite loss was a major limitation. The AWGC recommends assessment of appetite using validated instruments, such as the Simplified Nutritional Appetite Questionnaire [28], whereas the present study inferred appetite loss from gastrointestinal symptoms documented in the SGA interview. Although the SGA is a structured nutritional assessment, this approach has not been validated as a substitute for an appetite-specific questionnaire. In addition, gastrointestinal symptoms in ADHF may reflect intestinal congestion, edema, or acute illness rather than cachexia-related anorexia. Therefore, misclassification of the appetite component may have occurred and could have diluted the observed association between AWGC-defined cachexia and clinical outcomes.

Second, weight and BMI on admission may have been influenced by congestion and fluid overload. Although the AWGC weight-related criterion was applied using available clinical information, acute fluid retention in ADHF may mask tissue loss or distort BMI-based classification. Thus, cachexia diagnosed during acute hospitalization should be interpreted as a pragmatic clinical phenotype rather than as a definitive measure of chronic tissue wasting.

Third, CRP was measured once on admission and may have reflected both chronic cachexia-related inflammation and transient acute inflammatory responses related to congestion, infection, or acute physiological stress. Therefore, the CRP component should not be interpreted as a pure pathophysiological marker of chronic cachexia biology in this setting.

Fourth, the sample size was small and only 23 deaths occurred during follow-up. To reduce the risk of overfitting, we used a parsimonious Cox model adjusted for age, sex, and BNP, selected a priori on clinical grounds. However, residual confounding by nutritional status, anemia, renal function, physical function, medication use, recent heart failure hospitalization, and other unmeasured factors cannot be excluded. Therefore, the present findings should be interpreted as exploratory and hypothesis-generating rather than as evidence of a causal relationship.

Fifth, complete-case analysis and exclusion of patients who died during hospitalization may have introduced selection and survivorship bias. Patients who died in hospital were excluded because post-discharge outcomes could not be evaluated; however, this approach may have removed the sickest patients and may have underestimated the true prognostic impact of cachexia in the overall ADHF population.

Sixth, readmission was analyzed as the time to first all-cause readmission. Because readmission after ADHF is recurrent and multifactorial, this approach may have underrepresented the total burden of rehospitalization. Future studies should consider recurrent-event analyses, cause-specific readmission outcomes, and competing-risk approaches.

Despite these limitations, the observed association between AWGC-defined cachexia and mortality was biologically plausible, directionally consistent with prior literature, and accompanied by a clinically coherent vulnerability profile characterized by older age, lower BMI, poorer nutritional status, anemia, and reduced physical function. Nevertheless, larger multicenter studies with standardized appetite assessment, repeated inflammatory measurements, and more comprehensive adjustment are needed to validate these findings.

## 5. Conclusions

Cachexia on admission was associated with an increased risk of all-cause mortality within one year after discharge among older patients hospitalized for ADHF. These findings should be considered exploratory and require confirmation in larger longitudinal studies before the prognostic value of cachexia can be established.

## Figures and Tables

**Table 1:** Patient characteristics.

	Missing Data, *n* (%)	Overall	Non-Cachexia	Cachexia	*p*-Value
		(*n* = 78)	(*n* = 52)	(*n* = 26)	
Age, years	0	85 [80–90.5]	84 [79.75–88]	87.5 [81.75–92]	0.04
Male, *n* (%)	0	45 (57.7)	34 (65.4)	11 (42.3)	0.05
BMI, kg/m^2^	3 (3.8)	22.5 [20.2–25.8]	25.1 [22.5–27.0]	19.2 [18.1–20.5]	<0.001
NYHA Class III or IV, *n* (%)	0	48 (61.5)	33 (63.5)	15 (57.7)	0.62
LVEF, %	3 (3.8)	61 [29.625–64]	61 [35–63]	61 [23–70]	0.54
Long-term care insurance certification, *n* (%)	0	40 (50.6)	25 (48.1)	15 (57.7)	0.42
History of heart failure hospitalization, *n* (%)					
0		27 (34.6)	20 (38.5)	7 (26.9)	
1		33 (42.3)	24 (46.2)	9 (34.6)	
≥2		18 (23.1)	8 (15.4)	10 (38.5)	0.074
History of heart failure hospitalization within 6 months, *n* (%)	0	15 (19.2)	5 (9.62)	10 (38.5)	0.02
Comorbidities					
Hypertension, *n* (%)	0	49 (62.8)	32 (61.5)	17 (65.4)	0.74
Dyslipidemia, *n* (%)	0	12 (15.4)	4 (7.7)	8 (30.8)	0.007
Diabetes mellitus, *n* (%)	0	21 (27)	13 (25)	8 (30.8)	0.58
CKD, *n* (%)	0	28 (35.9)	17 (32.7)	11 (42.3)	0.4
Previous myocardial infarction, *n* (%)	0	9 (11.5)	6 (11.5)	3 (11.5)	1
Arrhythmia, *n* (%)	0	51 (65.4)	35 (67.3)	16 (61.5)	0.61
Musculoskeletal disorders, *n* (%)	0	18 (23)	11 (21.2)	7 (26.9)	0.57
Cancer, *n* (%)	0	11 (14.1)	6 (11.5)	5 (19.2)	0.36
Medications					
ACE or ARB, *n* (%)	3 (3.8)	19 (25.3)	13 (26.5)	5 (19.2)	0.28
MRA, *n* (%)	3 (3.8)	3 (4.2)	3 (6.1)	0 (0)	0.47
β-blockers, *n* (%)	3 (3.8)	42 (58.3)	30 (61.2)	14 (53.8)	0.08
Diuretics, *n* (%)	3 (3.8)	62 (82.7)	42 (85.7)	20 (70)	0.01
CCBs, *n* (%)	3 (3.8)	15 (20)	11 (22.4)	4 (15.4)	0.43
Antiarrhythmic drugs, *n* (%)	3 (3.8)	9 (12)	6 (12.2)	3 (11.5)	0.34
Anticoagulant, *n* (%)	3 (3.8)	48 (64)	28 (57.1)	20 (77)	0.05
SGLT-2 inhibitor, *n* (%)	3 (3.8)	14 (18.7)	9 (18.4)	5 (19.2)	0.68
Antiplatelet drugs, *n* (%)	3 (3.8)	12 (16)	10 (20)	2 (7.7)	0.14
ARNI, *n* (%)	3 (3.8)	17 (22.7)	10 (20)	7 (26.9)	0.46
Laboratory data on admission					
CRP, mg/dL	0	0.64 [0.2–2.3]	0.5 [0.2–1.8]	0.8 [0.2–4.3]	0.29
eGFR, mL/min/1.73 m^2^	0	39 [31–53]	43.5 [32–59]	35 [30–44]	0.07
BUN, mg/dL	0	25.4 [18.9–34.5]	24.9 [19–31]	26.6 [19–38]	0.34
Sodium, mEq/L	0	140 [138–142]	140 [138–142.5]	139.5 [136–142]	0.31
BNP, pg/mL	0	547 [271–1012]	527 [267–933]	547 [413–1091]	0.34
Albumin, g/dL	0	3.4 [3.1–3.8]	3.6 [3.2–3.9]	3.2 [3–3.7]	0.006
Hemoglobin, g/dL	0	11.5 [9.5–13.1]	12.1 [10.5–13.4]	10.1 [8.6–12]	0.004
Physical function					
Grip strength, kg	0	16.5 [13.5–20.5]	18.0 [15.2–24.3]	14.2 [9.4–16.7]	0.001
Arm circumference, cm	8 (10.3)	23 [20.5–26.1]	24.5 [22.6–27.4]	21.0 [19.2–23]	<0.001
Calf circumference, cm	8 (10.3)	29.5 [26.6–32.3]	31 [27.6–33]	27 [25.3–29.5]	<0.001
All-cause mortality within 1 year after discharge, *n* (%)	0	23 (29.5)	11 (21.2)	12 (46.2)	0.03
Readmission within 1 year after discharge, *n* (%)	0	32 (41)	21 (40.1)	11 (44)	0.76
Readmissions for heart failure within 1 year of discharge, *n* (%)	0	24 (30.8)	16 (30.8)	8 (30.8)	1.00

Data are presented as the median [interquartile range] or as *n* (%). ACE, angiotensin-converting enzyme; ARB, angiotensin receptor blocker; ARNI, angiotensin receptor neprilysin inhibitor; BMI, body mass index; BNP, B-type natriuretic peptide; BUN, blood urea nitrogen; CCBs, calcium channel blockers; CKD, chronic kidney disease; CRP, C-reactive protein; eGFR, estimated glomerular filtration rate; LVEF, left ventricular ejection fraction; MRA, mineralocorticoid receptor antagonist; NYHA, New York Heart Association; SGLT-2, sodium/glucose cotransporter 2.

**Table 2:** Cox proportional hazards model of factors associated with all-cause mortality within one year after discharge.

	HR	95% CI	*p*-Value
Cachexia	3.04	1.25–7.41	0.014
Age	1.01	0.96–1.07	0.709
Male	1.29	0.53–3.12	0.58
Log BNP	1.29	0.79–2.10	0.312

BNP, B-type natriuretic peptide; CI, confidence interval; HR, hazard ratio.

**Table 3:** Cox proportional hazards model of factors associated with all-cause readmission within one year after discharge.

	HR	95% CI	*p*-Value
Cachexia	1.65	0.74–3.71	0.223
Age	0.99	0.95–1.04	0.710
Male	0.92	0.43–1.96	0.824
Log BNP	0.84	0.55–1.28	0.415

BNP, B-type natriuretic peptide; CI, confidence interval; HR, hazard ratio.

## Data Availability

The data presented in this study are available on request from the corresponding author due to privacy and ethical restrictions.

## Supplementary Materials

The following supporting information can be downloaded at https://www.mdpi.com/article/10.3390/geriatrics11040098/s1, Table S1: Fulfillment and missingness of each AWGC cachexia component.
