# Supplementary material for: Association Between Cachexia Defined by the Asian Working Group for Cachexia Criteria and One-Year Post-Discharge Mortality in Older Adults with Acute Decompensated Heart Failure
Source: Geriatrics (Basel). 2026 Aug 4;11(4):98. doi: 10.3390/geriatrics11040098 (PMC13512389; doi:10.3390/geriatrics11040098)
Supplement: Supplementary file 1 [file geriatrics-11-00098-s001.zip › geriatrics-4408114-supplementary.pdf]

Supplementary Table S1. Fulfillment and missingness of each AWGC cachexia component.

Component	Missing data n (%)	Overall	Non-cachexia	Cachexia
		(*n* = 78)	(*n* = 52)	(*n* = 26)
Underlying chronic disease, n (%)	0	78 (100)	52 (100)	26 (100)
Weight loss >2% in 3–6 months, n (%)	0	11 (14.1)	4 (7.6)	7 (26.9)
BMI <21 kg/m², n (%)	2 (2.6)	25 (32.1)	1 (1.9)	24 (92.3)
Weight-related criterion fulfilled, n (%)	0	31 (39.7)	5 (9.6)	26 (100)
Appetite loss, n (%)	0	17 (21.8)	7 (13.5)	10 (38.5)
Low handgrip strength, n (%)	0	68 (87.2)	43 (82.7)	25 (96.2)
CRP >0.5 mg/dL, n (%)	0	44 (56.4)	28 (53.8)	16 (61.5)
At least one clinical/laboratory criterion, n (%)	0	72 (92.3)	47 (90.4)	25 (96.2)
AWGC-defined cachexia, n (%)	2 (2.6)	26 (33.3)	0	26 (100)

BMI, body mass index; CRP, C-reactive protein; AWGC, Asian Working Group for Cachexia.
